# Effect of Rabbit Epididymal Antimicrobial Peptide, REHb**β**P, on LPS-Induced Proinflammatory Cytokine Responses in Human Vaginal Cells *In Vitro*

**DOI:** 10.1155/2012/782019

**Published:** 2012-03-14

**Authors:** K. V. R. Reddy, D. Sukanya, M. S. Patgaonkar, C. Selvaakumar

**Affiliations:** ^1^Division of Molecular Immunology, National Institute for Research in Reproductive Health (NIRRH), Indian Council of Medical Research (ICMR), Jehangir Merwanji Street, Parel, Mumbai 400 012, India; ^2^Department of Biotechnology and Bioinformatics, Padmashree Dr. D.Y. Patil University, CBD Belapur, Navi Mumbai 400 614, India

## Abstract

Antimicrobial peptides (AMP's) protect epithelial surfaces including epididymis against pathogens and play a key role in orchestrating various defensive responses. Recently, we have identified one such AMP, rabbit epididymal hemoglobin-*β* subuit (REHb*β*P) from the epididymal fluid of rabbit, *Oryctologus cuniculus*. The demonstration of a protective role of REHb*β*P in epididymal epithelial cells (EPEC's) led us to investigate: (1) the identification of LPS interactive domain in REHb*β*P, and (2) whether the REHb*β*P of rabbit origin mediates vaginal cellular immune responses of another species (human). HeLa-S3, human vaginal epithelial cells (hVECs) were exposed to LPS or the LPS-stimulated cells treated with REHb*β*P or neutral peptide, *n*REHb*β*P. Effect of LPS and cytokines (IL-6 and IL-1*α*) and chemokines (IL-8, MCP-1) levels was determined in the culture supernatants. In response to the LPS, hVECs synthesized these mediators and the levels were significantly higher than controls. This enhancing effect was ameliorated when the LPS-induced hVECs were treated with REHb*β*P. Similar results were obtained on NF-*κ*B protein and hBD-1 mRNA expression. Confocal microscopy studies revealed that REHb*β*P attenuated the LPS-induced internalization of *E. coli* by macrophages. The chemotaxis studies performed using Boyden chamber Transwell assay, which showed elevated migration of U937 cells when the supernatants of LPS-induced hVECs were used, and the effect was inhibited by REHb*β*P. REHb*β*P was found to be localized on the acrosome of rabbit spermatozoa, suggesting its role in sperm protection beside sperm function. In conclusion, REHb*β*P may have the potential to develop as a therapeutic agent for reproductive tract infections (RTI's).

## 1. Introduction

A number pathogens can infect both male and female reproductive tracts in humans and animals [1]. In a large proportion of infections, products such as lipopolysaccharide (LPS) and endotoxins are responsible. LPS is a major structural and functional component of the outer membrane of Gram-negative bacteria [2] and exhibits a variety of toxic and proinflammatory activities. Therefore, identifying molecules that bind to LPS and neutralize its activity has clinical applications [3, 4]. 

The epididymis is anatomically connected to the urethra, so it is always at risk of ascending microbial invasion. It has been reported that in men the penile urethra is the entry for various STI-causing pathogens such as *Neisseria gonorrhoeae* and *Chlamydia trachomatis*, and urethritis is the most common clinical syndrome [5]. Infection originating from retrograde ascent of pathogens via the ejaculatory ducts, vas deferens, or the blood vessels supplying the epididymis is a common cause of acute epididymitis. Epididymitis is the most common intrascrotal inflammation and is a significant cause of urological consults [5, 6]. 

Epididymal epithelial cells (EPECs) form a barrier to create a unique microenvironment in the lumen, where interactions between EPECs and spermatozoa take place via the fluid milieu [7]. Spermatozoa binds a variety of proteins as they pass through the epididymis [8]. Beside sperm maturation, the epididymis is known to contain efficient self-defense machinery to deal with microbial infections [9, 10]. Recent studies have indicated that EPECs of rats, mice, and humans synthesized a variety of molecules having antimicrobial activities. Some of them are Bin-1*β* [11], Eppin [10], HE2-*α* [12], *β*-defensins [13], SOB3 [14], EP2/HE2 [5], hCAP-18 [15], and cystatin-11 [16]. It has also been reported that several hemoglobin- (Hb-) derived peptides of female reproductive tract of human [17–21] are effective against various sexually transmitted infections (STI's) causing pathogens.

Our group has recently isolated one such Hb-derived AMP, rabbit epididymal hemoglobin beta peptide (REHb*β*P) from rabbit epididymal fluid [22]. REHb*β*P showed 96% sequence identity with the human Hb-*β*  subunit. The purpose of the present study was to predict the LPS binding aminoacids in the REHb*β*P sequence. A further purpose was to evaluate the LPS-neutralizing activity of REHb*β*P by documenting its ability to block LPS-induced proinflammatory responses by LPS-sensitive target cells *in vitro*. In response to LPS induction, human vaginal epithelial cells (hVECs) produced elevated levels of various cytokines/chemokines (IL-8, IL-6, IL-1*α*, and MCP-1). This enhancing effect of LPS on these inflammatory biomarkers was ameliorated by the treatment of hVECs with REHb*β*P.

## 2. Material and Methods

### 2.1. Media and Reagents

Two 29 mer linear peptides REHb*β*P (104–133 amino acids) and *n*REHb*β*P (13–41 amino acids) of Hb-*β* subunit were commercially procured from USV Ltd., Mumbai, India. LPS-*E. coli *055:B5, N-formyl-methionyl-leucyl-phenylalanine (fMLP), and primary and secondary antibodies used in various experiments were procured from Sigma, USA. Kits for interleukin-6 (IL-6), interleukin-8 (IL-8), macrophage chemotactic protein-1 (MCP-1), and interleukin-1*α* (IL-1*α*) were obtained from R&D Systems, USA. For the generation of stock solutions, all reagents were dissolved in endotoxin-free water (Sigma, USA). All other reagents were purchased from Sigma-Aldrich (St. Louis, USA) unless specified otherwise.

### 2.2. Human Vaginal Epithelial Cells, Macrophages, and U-937 Cells

Human vaginal epithelial cells (HeLa-S3), macrophages, and U-937 cells were procured from National Center for Cell Sciences (NCCS), Pune, India, and grown in DMEM (HeLa-S3 cells) or RPMI-1640 (macrophages and U937 cells) as per the supplier's instructions.

### 2.3. Structure Prediction of REHb*β*P and Its Docking with LPS by Using Homology Modeling

The amino acid sequence of Hb protein was used for the selection of the REHb*β*P template sequence. Two main criteria were considered for the selection of the template: (i) the Hb protein should possess an authenticated crystal structure and (ii) the selected template must demonstrate maximum homology with the Hb protein. After selecting the template by the above criteria, Hb crystal structure was used to model the structure of REHb*β*P. Before this, the obtained template was energy minimized using Swiss pdb viewer [23] to remove steric clashes from the crystal structure by using WHATIF server (http://swift.cmbi.ru.nl/servers/html/index.html). To predict the overall stability, we first determined phi-psi angles followed by structural analysis using Ramanchandran plot and PROSA software, respectively [24]. Finally, the template was validated with What If server (http://swift.cmbi.ru.nl/servers/html/index.html). The peptide, REHb*β*P and the charged LPS were docked by using Hex 6.1 software [25]. While docking, for correlation type, we selected shape and electrostatic parameters and 3D LITE in FFT mode. Postprocessing was carried out using MM minimization. Of the generated docked structures, the least binding energy was selected. Binding interactions of the duo was visualized using chimera software.

### 2.4. Design and Synthesis of Peptides (REHb*β*P and nREHb*β*P)

The 15 mer sequence corresponding to 110–124 amino acids (LVIVLSHHFGKEFTP) of the Hb-*β*  subunit has been subjected to various bioinformatics tools to identify the aminoacids that are interactive with the LPS shown in Figure 2. A 29 mer sequence corresponding to 104–133 amino acids of the Hb-*β* subunit (RLLGNVLVIVLSHHFGKEFTPQVQAAYQK), which consists of the above 15 mer sequence, has been synthesized in circular form. To analyze the LPS binding specificity of REHb*β*P, a 29 mer non-LPS binding region corresponding to 13–41 amino acids (ALWGKVNVEEVGGEALGRLLVVYPWTQRF) of the Hb-*β* subunit has been synthesized and named as scrambled (control) or neutral peptide (*n*REHb*β*P), and its LPS binding and neutralizing activity was assayed along with REHb*β*P. The purity of these peptides was confirmed by mass spectrometry and amino acid analysis and found to be ~95%.

### 2.5. Cell Culture and Treatment

The hVECs used in this study constitutively expressed TLR4 and responded to the LPS [26]. On the day of treatment, cells at 70–80% confluence were passaged. In our previous study, we have demonstrated that LPS with concentrations of 10 *μ*g/mL did not inhibit the viability human endocervical epithelial cells (End1/E6E7) [27]; therefore, this dose was selected for this study.

The hVECs were seeded at a density of 2 × 10^6^ cells/well in 24-well plates and incubated for 24 h at 37°C in 5% CO_2_ and 95% air and divided into six groups: (1) cells grown in culture medium for 24 h without any treatment (medium control), (2) cells treated with REHb*β*P (60.61 *μ*M for 1 h), (3) scrambled peptide (*n*REHb*β*P) (60.61 *μ*M for 1 h), (4) cells induced with LPS alone (10 *μ*g/mL for 6 h), (5) after washing cells treated with REHb*β*P (60.61 *μ*M for 1 h), and (6) *n*REHb*β*P (60.61 *μ*M for 1 h). In addition to these six groups, we also included an additional group for chemotaxis experiments wherein the hVECs were treated first with polyclonal anti-TLR4 antibody before stimulation with LPS. At the end of treatment, cells were washed twice with PBS (pH 7.4), cultured for an additional 24 h, and the spent media along with cells from control and treated groups were collected for various studies.

### 2.6. ELISA to Determine REHb*β*P Binding to LPS

The ability of REHb*β*P to bind to LPS was determined by the method described earlier [27]. Briefly, a 96-well microtiter plate was coated with 100 *μ*L of LPS (5 *μ*g/mL) dissolved in PBS and incubated for 60 min with 100 *μ*L of two-fold serially diluted peptides (1.89–60.61 *μ*M). ELISA was developed with an affinity-purified rat antiserum raised against REHb*β*P and *n*REHb*β*P, and a secondary goat anti-rat antibody (Sigma, USA) conjugated to horse radish peroxidase-HRP. O-phenylenediamine (1 mg/mL) was used as a substrate, and the absorbance was measured at 490 nm on a microplate reader (ELX-800, Bio-Tek Instruments, and USA). A known peptide, *Scylla serrata*, antilipopolysaccharide factor-24 (SsALF-24) was used as positive control for LPS binding [27].

### 2.7. Measurement of Cytokine Levels by ELISA

Biomarkers of inflammation, namely, interleukin-6 (IL-6), interleukin-8 (IL-8), monocyte chemoattractant protein-1 (MCP-1), and interleukin-1*α* (IL-1*α*) were measured in all the six groups of culture supernatants by using commercially available human cytokine kits with matched antibodies (R&D system, USA) as described earlier [28]. Briefly, supernatants were cleared of cells by centrifugation at 1000 ×g for 10 min at 4°C and used for the estimation of cytokines (IL-6 and IL-1a), and chemokines (MCP-1 and IL-8) after determining the total protein concentration [29]. Compounds (LPS, REHb*β*P, and *n*REHb*β*P) interference with cytokine detection was ruled out by spiking known amounts of recombinant IL-6 and IL-8 and measuring the percentage cytokine recovery from compound-supplemented medium versus plain medium control.

### 2.8. Determination of Phospho-NF-*k*B p65 Levels by ELISA

To address the question of whether REHb*β*P competed with LPS at the receptor level or it affects occurred downstream from TLR4-LPS signaling, and to accomplish this task, the treatment protocol followed is the same as described under cell culture and treatment in Section 2. Preincubation of hVECs with the LPS ensured LPS-TLR binding in the absence of REHb*β*P, and hVECs stimulated with LPS alone ensured interaction between TLR4-LPS in the absence of REHb*β*P. The LPS-induced cells (group-4) were treated with REHb*β*P (group-5) or *n*REHb*β*P (group-6) for 1 h. After the treatment, hVECs were lysed with hypotonic HEPES lysis buffer (pH 7.4) and centrifuged at 1000 ×g for 10 min at 4°C. Supernatants were collected and total protein concentration was determined [29] before estimating NF-*k*B levels by ELISA as described earlier [30].

### 2.9. RT-PCR Analysis of hBD-1 Gene in hVECs

To determined if LPS induces the expression of human-beta defensin-1 (hBD1) mRNA, hVEC cells were seeded at a density of 10^6^ per well in 6-well plates. After being washed, cellular RNA was extracted by TRIZOL solution (Invitrogen) according to the manufacturer's protocol. The primer sequences used for hBD1 (196 bp) used were sense 5′-CTCTGCTTGCTGCCATTCTC-3′ and antisense 5′-AATCGTCTGCAAGTACA GGACAC-3′, and for GAPDH (199 bp) sense 5′-CCATTCATTGACCTCCACTACA-3′, antisense 5′-CGTTGCTGACAAT CTTGAGAGA-3′. Products of the expected sizes were generated with and separated on a 2% agarose gel with electrophoresis and visualized by ethidium bromide staining under UV illumination. The gels were scanned by using a Gel Documentation System (Gel Doc 2000, Bio-Rad Laboratories) and intensity of the bands was quantified by “Quantity-One” software.

### 2.10. Mcrophage Phagocytic Assay

Gram-negative bacteria (*E. coli*) were labeled with fluorescein isothiocyanate (FITC) as described earlier [17]. Macrophages (1 × 10^6^) were stimulated with LPS (10 *μ*g/mL for 1 h), washed twice with RPMI, and treated with REHb*β*P or *n*REHb*β*P (60.61 *μ*M for 1 h). After two washings in RPMI to remove peptides, cells were incubated at 37°C with the cell suspensions of FITC-labeled *E. coli* (ratio of macrophages: *E. coli* 1 : 20) in a total volume of 1 mL in siliconized glass tubes. Macrophages not exposed to *E. coli* were handled identically to determine the background. After 30 min of incubation, 1 mL of ice-cold complete RPMI medium per mL was added and centrifuged (110 ×g, 8 min) to separate phagocytic cells from free bacteria. Cells were washed twice in complete RPMI. The internalized bacteria and surface-bound bacteria were visualized under FITC optics using a confocal laser scanning microscope LCSM (Zeiss, 510 Meta, and Germany). We included plain macrophages as appropriate negative control to rule out any nonspecific activity. The number of internalized labeled *E. coli *in the presence of REHb*β*P or *n*REHb*β*P was determined as phagocytic index (PCI). The PCI was defined as the number of bacteria-containing macrophages per high-power field (×63)/total number of cells in the field, expressed as a percentage. For quantification of the PCI for a given condition, at least 25 fields were sequentially examined.

### 2.11. Chemotaxis Assay

The effect of REHb*β*P on U937 cell migration was assessed by a chemotaxis assay that used cAMP-activated U937 cells as described earlier [31]. For this study, cultures from all the six groups along with anti-TLR4 group were used and the treatment protocol was the same as discussed under cell culture and treatment. LPS-induced cells were treated with REHb*β*P (15.50 and 60.61 *μ*M for 1 h). Cells treated with 700 mM chemotactic peptide (N-formyl-methionyl-leucyl-phenylalanine (fMLP)) in PBS/BSA and placed in the lower chamber and considered as a positive control for cell migration. Conditioned media was harvested 24 h later from all the treatment and control groups and used for the infiltration/migration of U937 cells. Briefly, U937 cells (5 × 10^4^ in 500 *μ*L of serum-free medium) were loaded into the upper chamber of the Boyden chamber Transwells (0.4-*μ*M pore size polycarbonate membranes). Lower chambers were loaded with spent media obtained from REHb*β*P, *n*REHb*β*P, and anti-TLR4 antibody-treated groups. Two concentrations of REHb*β*P (60.61 and 15.50 *μ*M) were added to lower chamber. The chambers were then incubated in a humidified CO_2_ incubator at 37°C for 3 h. Nonmigrated U937 cells remained on the upper chamber of the insert were removed by placing the insert into a sterile 24-well plate, and cells migrating across the membrane were fixed, stained with crystal violet, and counted directly in a phase contrast microscope (×40). The results were expressed as the percentage of chemotaxis obtained in response to a maximal stimulation with the fMLP chemoattractant (100%).

### 2.12. Indirect Immunofluorescence

The presence and distribution of REHb*β*P on rabbit spermatozoa were analyzed by indirect immunouorescence (IF). The spermatozoa was collected from cauda epididymis, washed thrice in PBS, then placed on poly-L-lysine-coated glass slides and fixed 3.7% paraformaldehyde in phosphate buffer (pH 7.4) for 10 mm. After washing in PBS, the nonspecific sites were blocked with normal goat serum. Slides were incubated overnight at 4°C with primary REHb*β*P antibody raised in rats (1/250). Preimmune rat sera were used as negative control. Slides were incubated overnight at 4°C with FITC-labeled goat anti-rat secondary antibody (1/1000) for 1 h at room temperature. In between each step, the slides were washed six times for 5 min in blocking solution. Slides were counter stained with propidium iodide (PI), cover slipped, and mounted with VECTASHIELD mounting medium (Vector Labs, USA) and visualized with a confocal laser scanning microscope-LCSM (Zeiss, 510 Meta, Germany). Images were digitalized using CCD digital camera and Image-Pro Express Software at the central equipment facility of NIRRH, Mumbai. 

### 2.13. Statistical Analysis

Data are expressed as mean and standard error of the mean (SEM) of at least three independent experiments. Differences between the groups were compared by a one-way analysis of variance with post hoc range test Bonferroni adjustment. Results were considered statistically significant for *P* < 0.05.

## 3. Results

### 3.1. Structure Prediction, Synthesis, and Docking of REHb*β*P with LPS

The results of the present study demonstrated that REHb*β*P showed 96% sequence homology with rabbit Hb-*β* subunit. Three-dimensional structure of this subunit was used as a template for modeling REHb*β*P by Modeller software. Hemoglobin-*β* (Hb-*β*) subunit was docked with LPS by using Hex 6.1 software. Interaction of rabbit Hb-*β* subunit with LPS was analyzed by obtaining the crystal structure of rabbit Hb-*β* protein from pdb id: 2RAO viewer. Energy was minimized and bumps were removed by using SwissPdb viewer and Whatif server (http://swift.cmbi.ru.nl/servers/html/index.html). This structure was docked with the LPS using Z-DOCK server (http://zdock.bu.edu/). Overall, 2000 poses were generated, which were ranked according to their interactions. The best pose was selected and studied for its interactions using molecular visualization tool, Swiss pdb viewer.

**Figure 1:**
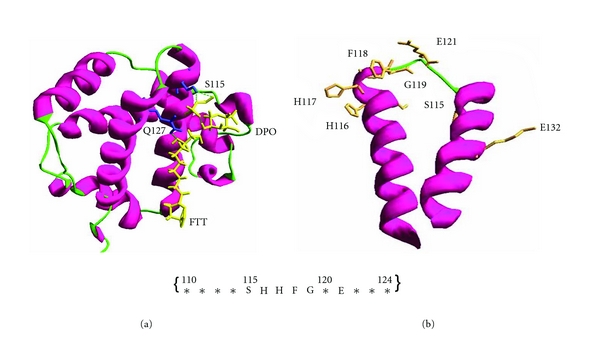
Hemoglobin-*β* (Hb-*β*) subunit docked with LPS. Few amino acids in between 110 to 124 of REHb*β*P showed interaction with LPS. The residues showing interaction are: Ser^115^, His^116^, His^117^, Phe^118^, Gly^119^, Glu^121^. The N-terminal and C-terminal region forms helical structure with a loop region in between ((a): 3D structure of Hb-*β* subunit; (b): interactive amino acids of REHb*β*P with the LPS are indicated).

Few amino acids (*****SHHFG*E***) within the 110 to 124 aminoacids of Hb-*β* subunit showed the interaction with LPS (Indian Patent filed on 18-10-2010). The residues showing interactions were Ser^115^, His^116^, His^117^, Phe^118^, Gly^119^, and Glu^121^(Figure 1). The N-terminal and C-terminal regions formed a helical structure with a loop region in between. Even though this short peptide comprises both helix and loop region, the binding was confined only to a loop region with the basic residues. The negatively charged phosphate ion showed interactions with the positively charged residues.

### 3.2. REHb*β*P Neutralizes LPS Activity

**Figure 2:**
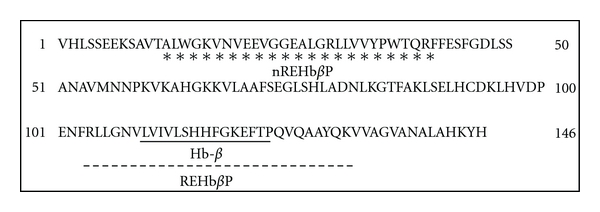
Translation of the rabbit Hb-*β* subunit coding sequence. Rabbit epididymal antimicrobial protein (REAMP), a 15 mer sequence (indicated in straight line) having antibacterial activity. This sequence corresponds to the amino acids 110–124 of Hb-*β* subunit (blue underline). Two 29 mer peptide sequences of rabbit epididymal Hb-*β* subunit REHb*β*P corresponding to the amino acids 104–133 (indicated in dotted line) and *n*REHb*β*P corresponding to the amino acids 13–41 (indicated in asterisks) of Hb-*β* subunit were synthesized. The *n*REHb*β*P has been considered as scrambled or control peptide.

**Figure 3:**
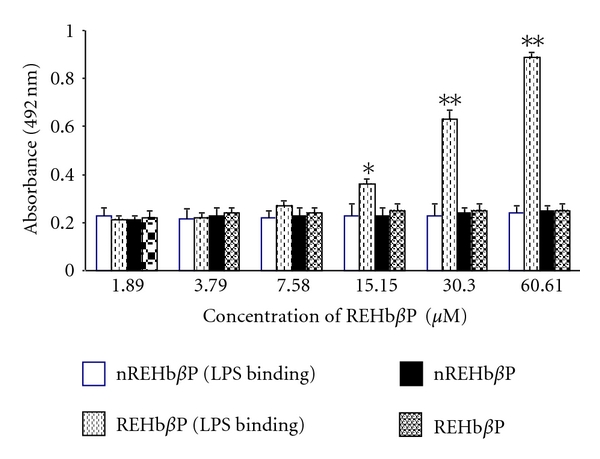
Determination of REHb*β*P binding to the LPS by ELISA. REHb*β*P bound to LPS in a dose-dependent manner. Maximum binding was observed with 60.61 *μ*M of peptide. No binding of *n*REHb*β*P to the LPS was observed. Values represent the mean ± S.D. of triplicate determinations performed on different days. Level of significance (*P < 0.05 and **P < 0.001 in compared to *n*REHb*β*P group) were calculated by ANOVA test followed by a post hoc Bonferroni analysis.

The peptide sequences synthesized are shown in Figure 2. REHb*β*P is able to bind and neutralized the LPS activity in a dose-dependent manner, with a higher binding at a concentration of 60.61 *μ*M. As expected, the scrambled peptide, *n*REHb*β*P, failed to bind and neutralize the LPS, whereas the positive control peptide, SsALF-24, significantly neutralized the LPS activity (Figure 3).

### 3.3. Suppressive Effect of REHb*β*P on Chemokine/Cytokine Production Is Dependent on Binding of REHb*β*P to LPS

**Figure 4:**
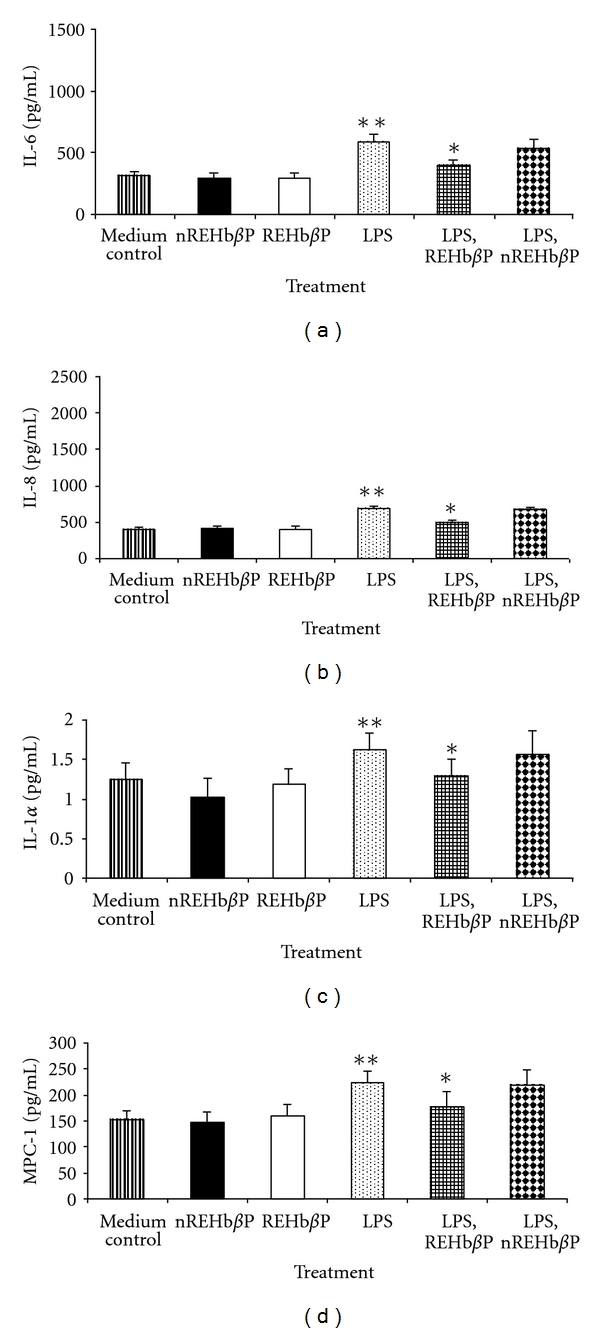
(a–d) The levels of cytokines/chemokine in the supernatants of hVECs. Cells were seeded at a density of 10^6^/well in a 24-well plates and treated with LPS (10 *μ*g/mL for 6 h), or LPS-induced (10 *μ*g/mL for 6 h) cells were treated with REHb*β*P (60.61 *μ*M for 1 h) or scrambled peptide, *n*REHb*β*P (60.61 *μ*M for 1 h), as detailed in Section 2. At the end of treatment, supernatants were collected and analyzed for inflammatory mediators by ELISA. REHb*β*P attenuated LPS-induced production of inflammatory mediators as compared with the cells that were treated with scrambled peptide. Values represent the mean ± S.D of triplicate determinations performed on different days. Level of significance (*P < 0.05 compared with LPS-induced group, **P < 0.05 compared with medium control) were calculated by ANOVA test followed by a Bonferroni analysis.

To investigate whether REHb*β*P has any effect on the LPS-induced synthesis/release of cytokine/chemokines, we measured the inflammatory biomarkers using ELISA in the culture supernatants as detailed in Section 2. LPS certainly promoted the secretion of cytokines/chemokines in hVECs. Figures 4(a)–4(d) illustrate significantly increased (*P* < 0.05) levels of cytokines (IL-6 and IL-1*α*) and chemokines (IL-8 and MCP-1) of hVECs when stimulated with nontoxic dose of LPS (10 *μ*g/mL for 6 h). In sharp contrast, LPS-induced cytokine production was dramatically suppressed and reached the baseline level when these cells were treated with REHb*β*P as compared with medium control (Figure 4). As expected, *n*REHb*β*P did not inhibit LPS-induced triggering of cytokine/chemokine production. The observed decrease of these mediators is not due to cytotoxicity, since REHb*β*P, *n*REHb*β*P, and LPS were nontoxic in the immunosuppressive dose range. Known concentrations of IL-8 and MCP-1 spikes were fully recovered (data not shown), thus observed results are not due to the assay interference.

### 3.4. REHb*β*P Downregulates LPS-Induced NF-*k*B Levels

**Figure 5:**
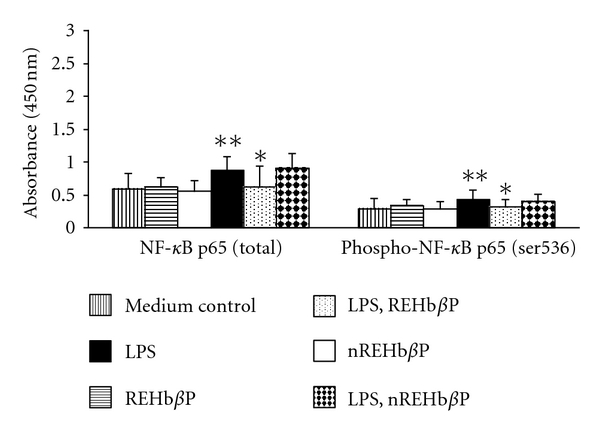
The levels NF-*k*B in hVEC evaluated by ELISA. Cells were seeded at a density of 10^6^/well in a 24-well plates and induced with LPS (10 *μ*g/mL for 6 h) or LPS-induced cells were treated with REHb*β*P (60.61 *μ*M for 1 h) or scrambled peptide *n*REHb*β*P (60.61 *μ*M for 1 h). At the end of treatment, cells were collected and lysates were prepared and analyzed for NF-*k*B. Level of significance (*P < 0.05 compared with the LPS-induced group, **P < 0.05 compared with medium control) were calculated by ANOVA test followed by a Bonferroni analysis.

The ELISA results demonstrated that induction of hVECs by LPS led to the upregulation of NF-*k*B. Interestingly, treatment of hVECs with REHb*β*P resulted attenuation of NF-*k*B activity in comparison with medium control (Figure 5).

### 3.5. LPS-Induced hBD1 mRNA Expression Is Inhibited by REHb*β*P

**Figure 6:**
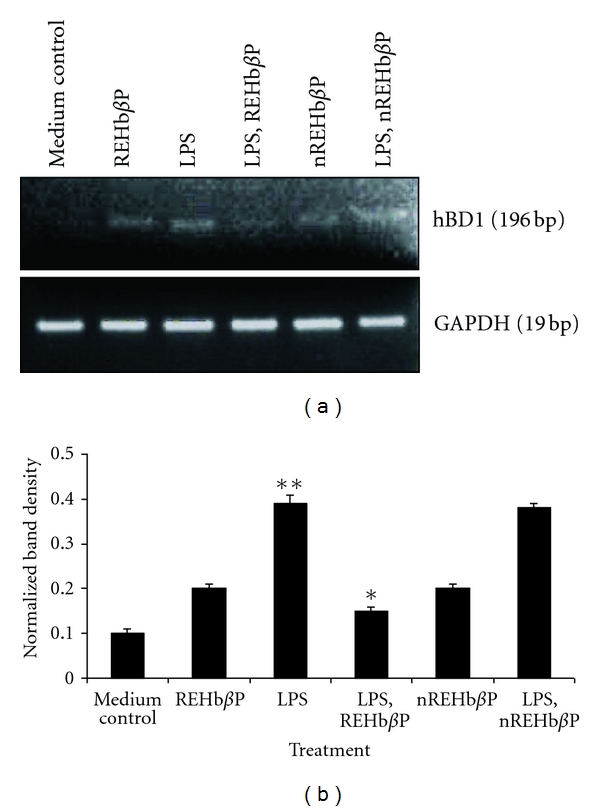
RT-PCR analysis of hBD-1 gene expression in hVECs. Cells were seeded at a density of 10^6^/well in a 24-well plates and treated with LPS (10 *μ*g/mL for 6 h) or LPS-induced (10 *μ*g/mL for 6 h) cells were treated with REHb*β*P (60.61 *μ*M for 1 h) or scrambled peptide (60.61 *μ*M for 1 h). At the end of treatment, cells were collected and lysates were prepared and analyzed for hBD1 and GAPDH mRNAs by RT-PCR as detailed in Section 2. (a) Expression of hBD1 mRNA was upregulated in LPS-induced cells and attenuated following the treatment of LPS-induced cells with REHb*β*P with respect to the scrambled peptide. Values are calculated as the mean ± SD of triplicate determinations. Representative image of RT-PCR analysis of hBD-1 mRNAs expression is shown. GAPDH blots confirmed roughly equivalent loading of RNA samples. (b) A quantitative assessment of the intensity of the each band was determined by densitometry. Levels of significance (*P < 0.001 compared with LPS-induced groups, **P < 0.001 compared with medium control) were calculated by ANOVA test followed by a Bonferroni analysis.

To define more fully the pattern of hVEC responsiveness to LPS and to examine whether the increased cytokine levels in hVECs were associated with any that occur in hVEC immune protection. For this, we chose hBD-1, (a known marker for cell protection against pathogens) and determined its mRNA expression after LPS induction in hVECs by RT-PCR. The results revealed that hVECs expressed hBD1 mRNA. By comparing these levels with that of GAPDH in LPS-induced hVECs, we could show hBD1 mRNA expression is upregulated by ~50%, whereas this upregulation is significantly inhibited by REHb*β*P (~24%) (group-5) compared with the medium control (group-1) (Figure 6).

### 3.6. REHb*β*P Downregulated LPS-Induced Phagocytosis

**Figure 7:**
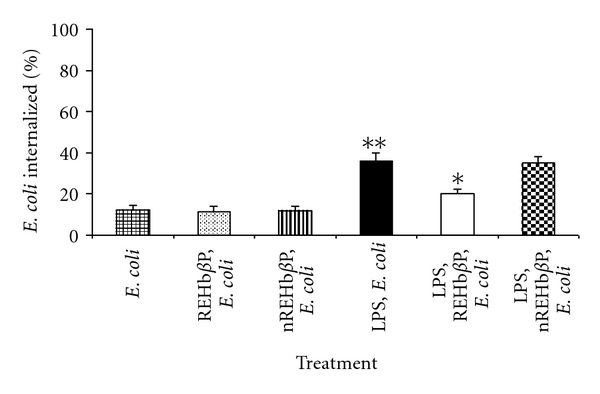
Macrophages when induced with LPS, phagocytosis index (PCI) was significantly upregulated over that of cells that were incubated alone either with* E. coli* or REHb*β*P or scrambled peptide. LPS-induced macrophages treated with REHb*β*P (60.61 *μ*M for 1 h) the number of *E. coli* internalized within macrophages was significantly reduced. Levels of significance (*P < 0.001 compared with the LPS-induced group, **P < 0.001 compared with *n*REHb*β*P group) were calculated by ANOVA test followed by a Bonferroni analysis.

We next investigated whether REHb*β*P modulate the LPS-induced phagocytic activity of macrophages. As shown in Figure 7, when the macrophages were treated with the LPS (10 *μ*g/mL for 1 h) (group-4), higher number of *E. coli* is internalized in the cytoplasm of the macrophages over that of cells that were incubated alone either with* E. coli* or REHb*β*P or scrambled peptide. The phagocytosis index (PCI) of medium control is ~1.79 ± 0.14%, *P* < 0.05, as compared to LPS stimulated cells (4.22 ± 0.70%). When LPS activated macrophages treated with REHb*β*P (group-5), the PCI is significantly reduced (2.31 ± 0.24%; *P* < 0.05). As expected, the scrambled peptide did not prevent the phagocytosis induced by LPS.

### 3.7. REHb*β*P Attenuated LPS-Induced Migration of U937 Cells

**Figure 8:**
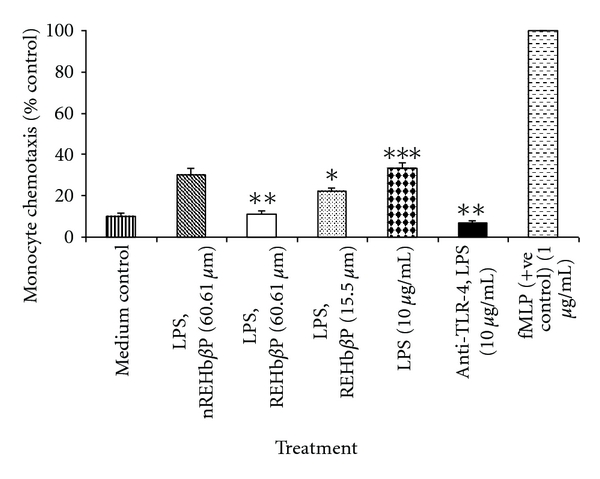
Determination of U937 cell migration/infiltration using chemotaxis assay. Culture supernatants of hVECs were collected after the cells were stimulated with LPS (10 *μ*g for 1 h). When these supernatants were placed in the lower Transwell chamber, U937 cell migration from upper Transwell was significantly increased over that of medium control. When supernatants obtained from LPS-induced cells that were treated with REHb*β*P (60.61 or 15.50 *μ*M for 1 h) and used in the lower Transwell chamber, the migration of U937 cells was reduced in a dose-dependent manner. REHb*β*P at a concentration of 60.61 *μ*M resulted higher inhibition of cell migration. As expected scrambled peptide did not suppress LPS-induced infiltration of U937 cell. The data was normalized to U937 chemotaxis in response to 0.1 mM fMLP in PBS/BSA, which was used to stimulate cell migration (100%). Each value is the mean ± S.D. of six individual observations obtained from three independent experiments. Levels of significance (*P < 0.05 compared with the LPS-induced group, **P < 0.001 compared with the LPS-induced group, ***P < 0.001 compared with the medium control) were calculated by ANOVA test followed by a Bonferroni analysis.

The effect of REHb*β*P on LPS-induced migration/infiltration of U937 cells was investigated* in vitro* by using a Boyden chamber Transwell assay. These results indicated that infiltration of U937 cells was more noticeable toward lower compartment containing conditioned medium obtained from LPS-induced hVECs compared with medium from cells that were induced with LPS followed by treatment with REHb*β*P (*P* < 0.001) and the effect was dose dependent with a higher reduction at 60.61 *μ*M concentration. This effect was directly proportional to the concentration of REHb*β*P tested. A ~12% reduction was observed with as low as 15.50 *μ*M concentration of REHb*β*P, whereas the maximum effect (~51%) was observed with 60.61 *μ*M. Pretreatment of U937 cells with anti-TLR4 antibody before LPS-induction significantly suppressed the migration of U937 cells. N-Formyl-Met-Leu-Phe was used as positive control for U937 cell chemoattraction and was considered as 100% (Figure 8).

### 3.8. REHb*β*P Localized on the Acrosome of Rabbit Spermatozoa

**Figure 9:**
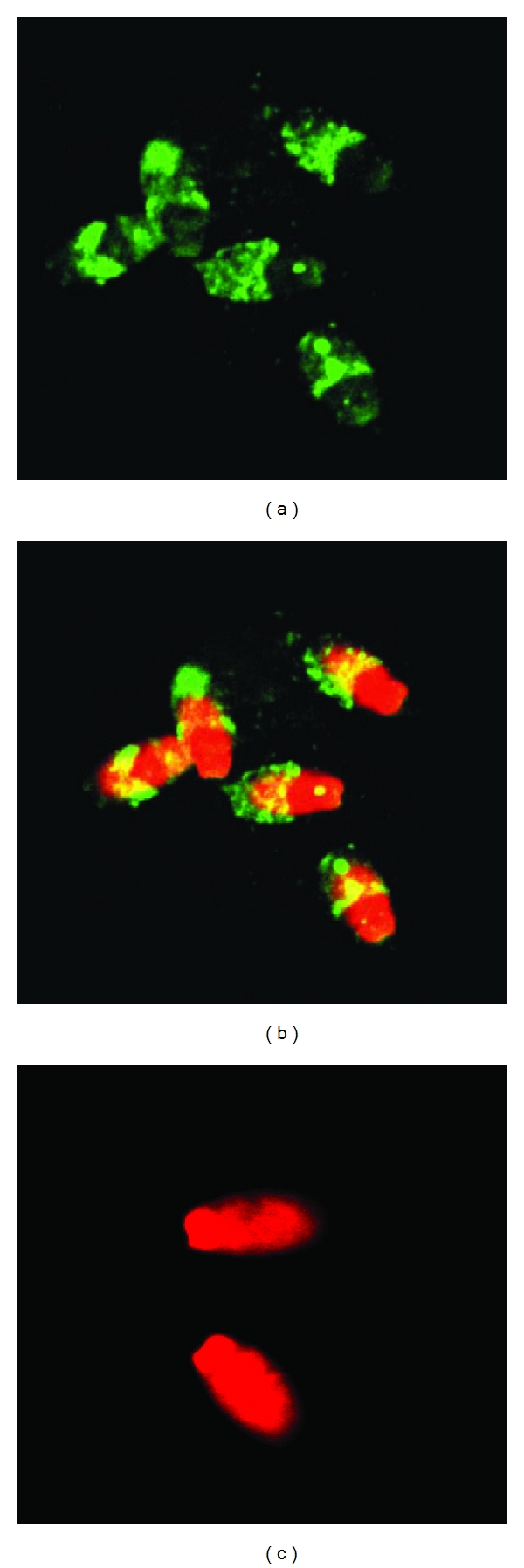
Images showing the immunolocalization of REHb*β*P on rabbit epididymal spermatozoa (Mag × 100; a–c) ((a): FITC; (b): PI, FITC merge and (c): preimmune serum control). REHb*β*P localized as a coat on the acrosome of sperm heads. The figures shown are the representative pictures from three independent experiments.

Next, we evaluated whether or not REHb*β*P is expressed by the rabbit epididymal spermatozoa. Immunofluorescence results revealed the presence of REHb*β*P-positive immunostaining on the sperm surface covering the entire acrosomal region of the sperm. No other region of the sperm appears to be positive for REHb*β*P (Figure 9).

## 4. Discussion

In recent years, an innate and adoptive immune function of reproductive tract to pathogens has gained a significant interest among scientists. The candidate players participating in the maintenance of epididymis homeostasis are just beginning to be emerged. The epididymis is anatomically connected to the urethra, hence always at risk of ascending microbial invasion. Infection originating from retrograde ascent of Gram-negative bacteria via the ejaculatory ducts and the vas deferens is a common cause of acute epididymitis, which sometimes leads to infertility [6]. 

Lipopolysaccharide (LPS) is a structural component of the outer membrane of nearly all Gram-negative bacteria and is an important protein against the permeability of bactericidal agents, including AMPs. In the present study by the use of a variety of biochemical and immunologic approaches, we describe interaction of the REHb*β*P with the LPS and its consequences on cellular immune responses.

By using Moeller software, the structure of REHb*β*P was acquired. Studies suggested that the interaction between LPS and the amphipathic loop of Hb-*β* subunit is of an electrostatic nature whereby the positive charges of the peptide are assumed to bind to the negative charged groups of LPS (phosphates and carboxylates). REHb*β*P binds specifically to LPS with increasing concentration. Contrary to REHb*β*P, the scrambled peptide, *n*REHb*β*P did not bind to LPS, suggesting neutralizing property of REHb*β*P.

 Therefore, identification of such molecules that bind and neutralize the toxic effects of pathogens may have clinical application as a therapy for the treatment of reproductive complications. REHb*β*P, identified in the rabbit epididymis, is one such peptide belongs to the Hb family of proteins. Recent evidence suggests that besides O_2_ transport, several Hb-derived peptides also perform defense functions [19, 20].

Given the diverse effects of LPS signaling in the inflammation process, we hypothesized that REHb*β*P intervenes in the interaction between LPS and its surface receptor TLR4 on hVECs. The effect of REHb*β*P on cytokine production/release by hVECs in response to the LPS was investigated in comparison with that of the scrambled peptide. Inflammatory mediators were chosen as the principal end points due to their established involvement in tissue inflammation, immunoregulation, and macrophage/neutrophil migration [32]. REHb*β*P attenuated the secretion of all the four biomarkers of inflammation, suggesting that inhibition involves cellular events that are NF-*k*B independent and occur downstream from NF-*k*B gene transactivation. Similar inhibitory effect of crab hemolymph derived peptide, SsALF-24 on cytokine/chemokine levels has been reported recently [30]. 

The above findings raise the important question of how REHb*β*P acts after the LPS has interacted with its receptor TLR4 on hVECs and inhibits LPS-induced biomarkers of inflammation. These unexpected results prompted us to speculate on the alternative mechanisms of REHb*β*P-mediated protection unrelated to the LPS binding to TLR4. One possible reason is that LPS-TLR4 interaction may activate cell surface receptors other than TLR4 through which REHb*β*P may exert its effects or such an interaction may facilitate the transfer of REHb*β*P signals intracellularly, and thereby inhibit synthesis/release of cytokines/chemokines. However, at present, it is not known the cellular targets for REHb*β*P and is the subject of current investigation in our laboratory. Recently, in an elegant study, Du et al. [19] have reported that Hb-derived peptides possess dual action centers, LPS recognition, and a peroxidase cycle (POX) activity sites. The latter produces reactive oxygen species (ROS), which recognize the LPS and neutralize it. REHb*β*P is one such Hb-derived AMP and perform similar functions.

In the epididymis, a number of cell types contribute to the local environment and secrete an array of AMPs, which protect spermatozoa during their epididymal transit. However, it is not known whether REHb*β*P perform similar function of sperm protection during maturation in the epididymis. To know this, one of the known epididymal AMPs, *β*-definsin-1 mRNA expression was determined and found significantly elevated after the induction of hVECs with LPS. Interestingly, REHb*β*P caused a significant blunting of LPS-induced activation of hBD-1 mRNA, attributing anti-inflammatory activity of REHb*β*P. These results were in agreement with previous report, where it was shown the upregulation of several AMPs of the defensins family by LPS both at mRNA and protein levels [6].

In view of the above observations, studies were further extended, which demonstrated the involvement of REHb*β*P in macrophage phagocytosis. It has been reported that recognition of bacteria by host cells depends on the receptor-ligand interaction. Upon recognition of pathogens, TLR4-LPS complex can transfer the signal into the host cell [33]. The present observations reveal that REHb*β*P inhibit phagocytosis of *E. coli *by inhibiting the LPS -TLR4 interactions. REHb*β*P, is a nonmyeloid cell-derived protein, its involvement in host defense has not been demonstrated previously. To our knowledge, this is the first report to document a potential role for REHb*β*P in cellular immune responses *in vitro*. Besides, we also demonstrated that the culture supernatants of LPS-induced hVECs show enhanced chemotaxis of U937 cells. In contrast, LPS-stimulated cells treated with REHb*β*P led to the inhibition of LPS-induced migration of U937 cell in a dose-dependant manner, confirming once again a protective role for REHb*β*P on hVECs.

Further, we analyzed whether REHb*β*P is present on the spermatozoa. The immunofluorescence revealed that REHb*β*P appears as a coat covering the acrosomal region of sperm head in rabbits. The localization suggests that REHb*β*P might be involved in some process of sperm maturation besides its antibacterial function. Cao et al. [6] have reported that rat caput epididymal-specific *β*-defensin peptide; Bin-1*β* plays dual roles in antibacterial and sperm motility. However, the exact role of REHb*β*P in sperm capacitation, acrosomal induction, sperm-egg recognition and binding is currently under investigation in our laboratory.

In conclusion, the aforementioned studies strongly reveal that REHb*β*P is capable of protecting epididymal sperm from pathogen-mediated insults. The expression pattern of REHb*β*P on rabbit spermatozoa implies complex biological functions beyond the immunoregulation. In pathological condition of epididymis like epididymitis, clarifying how pathogens regulate the expression of REHb*β*P is another interesting line of inquiry. These studies will aid in identifying therapeutic targets for the prevention and treatment of reproductive tract infections (RTIs).
